# Amorphous InGaZnO Thin-Film Transistors with Double-Stacked Channel Layers for Ultraviolet Light Detection

**DOI:** 10.3390/mi13122099

**Published:** 2022-11-28

**Authors:** Zenghui Fan, Ao Shen, Yong Xia, Chengyuan Dong

**Affiliations:** Department of Electronic Engineering, Shanghai Jiao Tong University, Shanghai 200240, China

**Keywords:** thin film transistor (TFT), amorphous InGaZnO (a-IGZO), double-stacked channel layers (DSCL), ultraviolet (UV) light, oxygen vacancy (V_O_), Technology Computer Aided Design (TCAD)

## Abstract

Amorphous InGaZnO thin film transistors (a-IGZO TFTs) with double-stacked channel layers (DSCL) were quite fit for ultraviolet (UV) light detection, where the best DSCL was prepared by the depositions of oxygen-rich (OR) IGZO followed by the oxygen-deficient (OD) IGZO films. We investigated the influences of oxygen partial pressure (P_O_) for DSCL-TFTs on their sensing abilities by experiments as well as Technology Computer Aided Design (TCAD) simulations. With the increase in P_O_ values for the DSCL depositions, the sensing parameters, including photogenerated current (I_photo_), sensitivity (S), responsivity (R), and detectivity (D*) of the corresponding TFTs, apparently degraded. Compared with P_O_ variations for the OR-IGZO films, those for the OD-IGZO depositions more strongly influenced the sensing performances of the DSCL-TFT UV light detectors. The TCAD simulations showed that the variations of the electron concentrations (or oxygen vacancy (V_O_) density) with P_O_ values under UV light illuminations might account for these experimental results. Finally, some design guidelines for DSCL-TFT UV light detectors were proposed, which might benefit the potential applications of these novel semiconductor devices.

## 1. Introduction

Ultraviolet (UV) light detectors have been extensively investigated in recent years due to their wide applications in flame detection, health motoring, environmental surveillance, and optical communication [1,2,3]. Amorphous oxide semiconductors (AOSs) are interesting materials for UV light detection because of their high transparency and wide band gap (E_g_ > 3 eV) [4,5,6]. Amorphous InGaZnO thin film transistors (a-IGZO TFTs) have especially attracted much attention as UV light detectors because of their great responsivity, small dark current, low manufacturing temperature, good uniformity, and outstanding electrical stability after bending [5,6,7,8,9,10]. In order to achieve higher UV light detectivity of a-IGZO TFTs, the researchers have reported several device improving methods, including choosing high-k gate dielectric materials, adding photosensitive absorption layers, adopting high work-function-difference capping layers, etc. [11,12,13]; on the other side, the optimization of processing conditions, e.g., using plasma enhanced atomic layer deposition (PEALD), adjusting oxygen partial pressure (P_O_) during preparations, etc., also seemed effective to make a-IGZO TFTs more sensitive to UV light illuminations [14,15,16,17]. 

Recently, we proposed to use double-stacked channel layers (DSCL) to increase the detecting abilities of a-IGZO TFT UV sensors, resulting in a good sensitivity of 28.6 dB when they were illuminated by 370 nm UV light [18]. In contrast to the optimized DSCL structure for general-purpose TFT devices [19], the best DSCL deposition consequence of oxygen-rich (OR) IGZO followed by oxygen-deficient (OD) IGZO was proven for UV light detection [18]. However, the detailed design methods as well as the related physical essence are still unknown for a-IGZO TFTs with DSCL (DSCL-TFTs) UV light detectors. 

In this study, the UV light response properties of the DSCL-TFTs using various P_O_ were comparatively investigated, with the related physical mechanisms demonstrated through experiments as well as Technology Computer Aided Design (TCAD) simulations. It was found that the UV light photogenerated current (I_photo_), sensitivity (S), responsivity (R), and detectivity (D*) of the DSCL-TFTs increased when P_O_ for the DSCL depositions became smaller. In addition, the back channel (OD-IGZO) was more sensitive to P_O_ variation than the front channel (OR-IGZO) under UV light illuminations. X-ray photoemission spectroscopy (XPS) and ultraviolet-visible spectrophotometer (UVS) measurements were employed to ascertain the variations of the defect density and light absorption with P_O_ for a-IGZO depositions. Finally, a qualitative model was proposed and elaborated to explain these experimental data through TCAD simulations.

## 2. Materials and Methods

**Figure 1:**
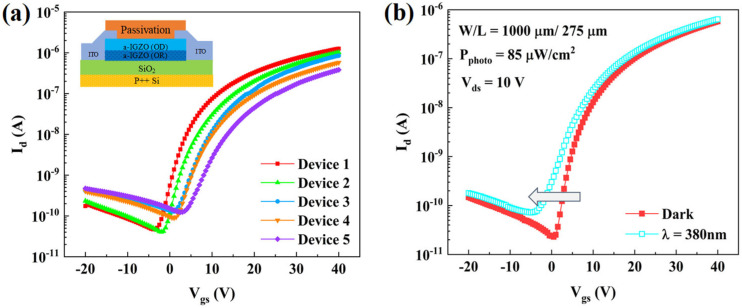
(**a**) Transfer curves of the DSCL-TFTs in dark, with the inset denoting the schematic cross-section of the five samples; (**b**) transfer curve evolution of Device 1 under 380 nm UV light illumination for 600 s.

Five samples of DSCL-TFTs (Device 1–5) were prepared on the p++ silicon wafers (gate electrodes) with 500 nm-thick SiO_2_ (gate insulators), with the schematic cross-section shown in the inset of Figure 1a. Among them, Device 2 was used as the reference sample. All the devices had the same structures but the processing conditions of their channel layers were different, as shown in Table 1. Both the OD-IGZO and OR-IGZO were deposited via magnetron sputtering, where the atomic ratio of the target was In: Ga: Zn = 1:1:1. After the DSCL depositions, the 200 nm-thick indium tin oxide (ITO) layers were prepared as source/drain (S/D) electrodes, followed by the 50 nm-thick SiO_2_ layers as passivation layers. Both the S/D electrodes and the passivation layers were also deposited by magnetron sputtering. Three shadow masks were used to pattern the DSCL, S/D electrodes, and passivation layers during sputtering, leading to the channel width/length of 1000 μm/275 μm, respectively. Finally, all the samples were annealed at 425 °C in N_2_ for 7200 s.

The electrical properties of the DSCL-TFTs were measured at room temperature (RT) using a Keithley 2636 semiconductor parameter analyzer (Solon, OH, USA). The 380 nm UV light illumination was achieved by the combination of a 150 W Xenon lamp, a monochromator, and an optical fiber. The incident optical power density was measured by an optical power meter (Thorlabs, PM100A, Newton, NJ, USA) with a Si photodiode sensor (Thorlabs, S120VC, Newton, NJ, USA), which was confirmed as 0.085 mW/cm^2^. The transmittance and the chemical bonding states of a-IGZO films were measured by a UV-visible spectrophotometer (EVOLUTION 300, Waltham, MA, USA) and an XPS analyzer (AXIS Ultra DLD, Manchester, UK), respectively. In order to thoroughly clean the sample surfaces, 10 nm-depth Ar-ion etching treatments were employed before recording the XPS spectra. Victory Device, one of the most popular TCAD tools from Silvaco, Inc., (Santa Clara, CA, USA) was used to investigate the physical essence relating the experimental results reported in this study [20].

## 3. Results and Discussion

Figure 1a shows the transfer characteristics of the five samples. One may notice that the transfer curves of the DSCL-TFTs shifted positively with P_O_ (for the channel-layer depositions) increasing which is consistent with other reports [21]. Interestingly, these curves were quite sensitive to the UV light illuminations. As shown in Figure 1b, the transfer curve of Device 1 apparently shifted in the negative direction when it was illuminated by the 380 nm UV light for 600 s. However, this sensitivity to the UV light illumination was different for the five samples, implying that the P_O_ values for the DSCL depositions evidently influenced the defect density in a-IGZO under UV light illuminations.

In order to quantitatively study the sensing properties of the DSCL-TFTs, we defined some useful terms, i.e., net photogenerated current (I_photo_), sensitivity (S), responsivity (R), and detectivity (D*) [4,18]. I_photo_ was defined as follows:

(1) $$I_{\mathrm{photo}}=I_{\mathrm{illuminated}}-I_{\mathrm{dark}}$$

where I_illuminated_ is the total photo current under illumination and I_dark_ is the dark current. In this study, I_dark_ was fixed as 10^−10^ A for all the measurements and I_illuminated_ was obtained under the same gate voltage corresponding to I_dark_.

Sensitivity (S), the ratio of the net photogenerated current and the dark current of the photodetectors, was defined as follows:

(2) $$S=20\mathrm{lg}\frac{I_{\mathrm{photo}}}{I_{\mathrm{dark}}}$$

Responsivity (R), the ratio of the net photogenerated current and the incident light power of the photodetectors, was defined as follows:

(3) $$R=\frac{I_{\mathrm{photo}}}{P_{\mathrm{light}}}$$

where P_light_ is the incident light power.

Detectivity (D*), a figure about the minimum detection of photodetectors, which was considered as the normalization of responsivity [16], was defined as follows:

(4) $$D*=\frac{R}{\sqrt{2eJ_{\mathrm{dark}}}}$$

where *e* is elementary charge and J_dark_ is the dark current density.

**Figure 2:**
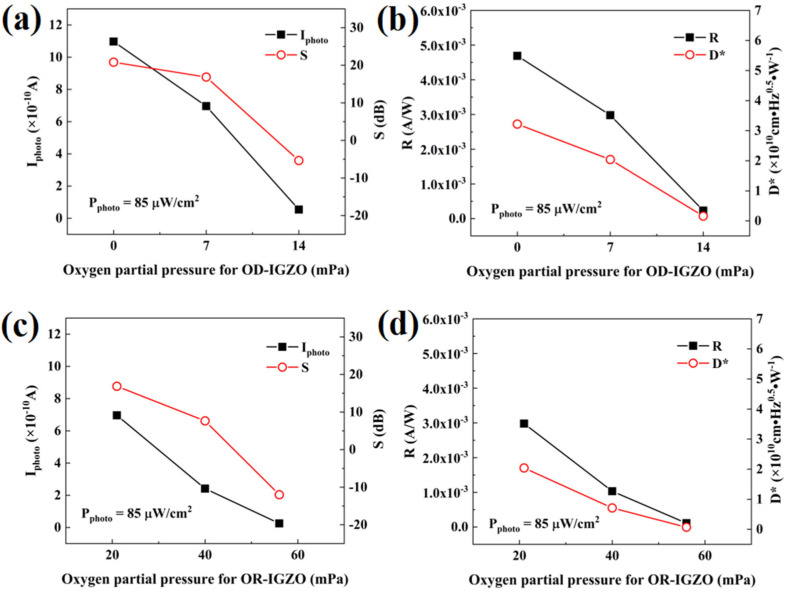
Dependence of the oxygen partial pressures for OD-IGZO on (**a**) I_photo_/S and (**b**) R/D* of the DSCL-TFTs; dependence of the oxygen partial pressures for OR-IGZO on (**c**) I_photo_/S and (**d**) R/D* of the DSCL-TFTs.

Figure 2a,b demonstrates the dependence of I_photo_, S, R, and D* on P_O_ for the OD-IGZO depositions. Here, the P_O_ values for OD-IGZO increased from 0 mPa to 14 mPa, while P_O_ for OR-IGZO was fixed at 21 mPa. On the other hand, Figure 2c,d shows the influence of P_O_ for the OR-IGZO depositions on I_photo_, S, R, and D*. Here, the P_O_ values for OR-IGZO increased from 21 mPa to 56 mPa, while P_O_ for OD-IGZO was fixed at 7 mPa. Obviously, all these parameters, including I_photo_, S, R, and D*, decreased with P_O_ increasing. In addition, for the purpose of analyzing the UV light sensing degradation of the DSCL-TFTs with the increase in P_O_ values, the variation ratio ΔI_photo_/ΔP_O_ was defined in this study (see Table 2). For the case of the P_O_ variation for OD-IGZO, ΔI_photo_/ΔP_O_ was −7.5 × 10^−11^ A·mPa^−1^. However, when the P_O_ value for OR-IGZO changed, ΔI_photo_/ΔP_O_ became −1.9 × 10^−11^ A·mPa^−1^. Similarly, when P_O_ for OD-IGZO increased, ΔS/ΔP_O_, ΔR/ΔP_O_, and ΔD*/ΔP_O_ were −1.9 dB·mPa^−1^, −3.2 × 10^−4^ A·W^−1^·mPa^−1^, and −2.2 × 10^9^ Jones·mPa^−1^, respectively. However, when P_O_ for OR-IGZO increased, ΔS/ΔP_O_, ΔR/ΔP_O_, and ΔD*/ΔP_O_ were −0.8 dB·mPa^−1^, −8.2 × 10^−5^ A·W^−1^·mPa^−1^, and −5.6 × 10^8^ Jones·mPa^−1^, respectively. We may draw a conclusion that the UV light response properties of the DSCL-TFTs were more sensitive to P_O_ variations for the back channel (OD-IGZO) than those for the front channel (OR-IGZO). These results are consistent with a recent publication, which indicated that the oxygen vacancies (V_O_) located at the back channel of the a-IGZO layer played an important role in UV light response [22]. This conclusion might lead to some new approaches to improving the DSCL-TFT UV light detectors by the modulation of P_O_ valves.

**Figure 3:**
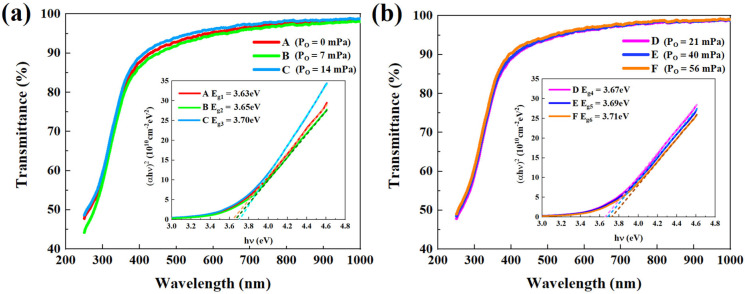
Transmittance spectra of (**a**) OD-IGZO and (**b**) OR-IGZO films; the insets show the fitting curves and the optical band gaps of OD-IGZO and OR-IGZO films, respectively. Here, the six samples (A, B, C, D, E, and F) were sputtered at P_O_ = 0, 7, 14, 21, 40, and 56 mPa, respectively.

To analyze the physical essence relating the abovementioned experimental results, we used a UV-visible spectrophotometer and an XPS analyzer to characterize the differences between the material properties of OD-IGZO and OR-IGZO. Figure 3 illustrates the transmittance spectra of OD-IGZO and OR-IGZO films, the corresponding Tauc plots, and linear fitting lines, which are shown in the insets. After obtaining the adsorption spectra from the transmittance spectra, we derived the optical energy band gaps by Tauc law using the following equation [19]:

(5) $${\left(\alpha h\upsilon \right)}^{2}=A\left(h\upsilon -E_{g}\right)$$

where α is the absorption coefficient, h is the Planck constant, hυ represents the photon energy, and A is a constant related to UV light adsorption. The value of E_g_ was determined by the intersection point of the linear fitting line and the x-axis. The overall transmittances of all the a-IGZO films were larger than 90% in the visible light region, though they quickly dropped when the light wavelength decreased below 400 nm, confirming the good UV light adsorption of a-IGZO films. As shown in Figure 3, both OD-IGZO and OR-IGZO exhibited almost the same optical bandgap values, i.e., around 3.7 eV. This suggests that P_O_ for a-IGZO depositions hardly influenced the UV light absorption of a-IGZO films in this study.

**Figure 4:**
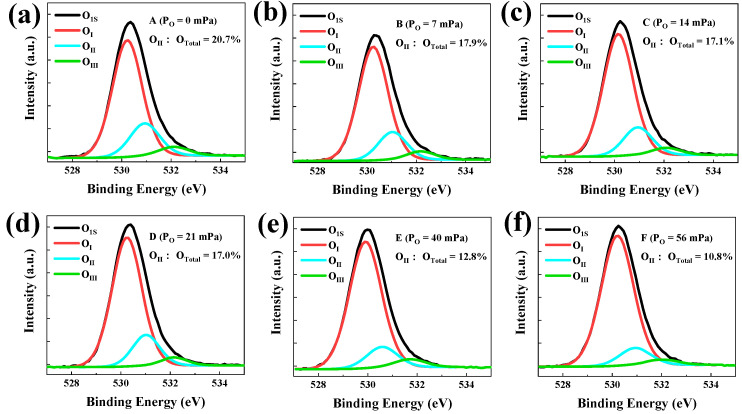
The XPS measurement and analysis results of the six a-IGZO films. Here, samples A (**a**), B (**b**), C (**c**), D (**d**), E (**e**), and F (**f**) were deposited at the oxygen partial pressure of 0, 7, 14, 21, 40, and 56 mPa, respectively.

Figure 4 depicts the XPS spectra of O1s signals in the six a-IGZO films (samples A, B, C, D, E, and F) prepared at various P_O_ values (0, 7, 14, 21, 40, and 56 mPa, respectively), corresponding to the channel-layer deposition conditions for the DSCL-TFTs shown in Figure 1. All the O1s spectra were calibrated by using the C1s (284.8 eV) as the reference [23,24,25,26], where the O1s peak was deconvoluted into three Gaussian fitting sub-peaks that approximately centered at 530.3 eV (O_I_), 531.0 eV (O_II_), and 532.2 eV (O_III_), respectively. The low binding energy O_I_ peak could be attributed to the oxygen bonds with metal, the high binding energy O_III_ peak might be related to the hydrated oxides defects, and the middle binding energy O_II_ peak was associated with oxygen vacancies (V_O_) [26]. In order to characterize the variations of V_O_ with the P_O_ values for IGZO deposition, the peak area ratio of the O_II_ over the total area of O1s peak (O_Total_ = O_I_ + O_II_ + O_III_) was calculated. One may notice that the area ratio O_II_/O_Total_ decreased from 20.7% to 10.8% when P_O_ increased from 0 mPa to 56 mPa. These results proved that the V_O_ density of the a-IGZO layer evidently decreased with P_O_ increasing during its deposition.

So far, three important results have been obtained from the aforementioned experiments: (1) The UV light response properties of the DSCL-TFTs evidently depended on the P_O_ value for the channel layer depositions; (2) The P_O_ variation for OD-IGZO at the top side of DSCL showed stronger influences on DSCL-TFT UV detectors than that for OR-IGZO at the bottom side of DSCL; (3) P_O_ hardly influenced the UV light absorption of a-IGZO films but evidently changed the defect density, especially the Vo density. To ascertain the related physical mechanisms, we employed TCAD simulations and analysis on the DSCL-TFTs illuminated by UV lights. Exponential and Gaussian functions were used to model the band tail states and deep states in a-IGZO films, respectively. Specifically, the donor-like tail states (g_TD_), the acceptor-like tail states (g_TA_), the donor-like deep states (g_GD_), and the acceptor-like deep states (g_GA_) were defined as Equations (6)–(9) [20]. Here, E_c_ and E_v_ is the conduction band edge energy and the valence band edge energy, respectively. The exact meanings of the corresponding parameters are all listed in Table 3.

(6) $$g_{\mathrm{TD}}(E)=\mathrm{NTS}\times \exp\left(\frac{E_{V}-E}{\mathrm{WTD}}\right)$$

(7) $$g_{\mathrm{TA}}(E)=\mathrm{NTA}\times \exp\left(\frac{E-E_{C}}{\mathrm{WTA}}\right)$$

(8) $$g_{\mathrm{GD}}(E)=\mathrm{NGD}\times \exp\left[-{\left(\frac{E-\mathrm{EGD}}{\mathrm{WGD}}\right)}^{2}\right]$$

(9) $$g_{\mathrm{GA}}(E)=\mathrm{NGA}\times \exp\left[-{\left(\frac{E-\mathrm{EGA}}{\mathrm{WGA}}\right)}^{2}\right]$$

As shown in Table 3, n(V_O_) is the initial Vo concentration and n(V_O_^2+^) represents the initial secondary ionized V_O_ concentration. For the simulations, there were two reactions to describe the ionization process in a-IGZO [20]:

(10) $$V_{O}^{x}=V_{O}^{••}+2e^{\prime }$$

(11) $$O_{i}^{\prime \prime }=O_{i}^{x}+2e^{\prime }$$

Here, $V_{O}^{x}$ and $V_{O}^{••}$ represent neutral oxygen vacancy and secondary ionized oxygen vacancy, respectively. Reaction (10) indicates that n(V_O_^2+^) should be affected by n(V_O_), which is related to P_O_ for a-IGZO deposition. Accordingly, n(V_O_^2+^) is indirectly influenced by the P_O_ value. It is generally believed that NGD is mainly associated with the V_O_ density [19,21]. Therefore, n(V_O_), n(V_O_^2+^), and NGD are all connected to the P_O_ values for a-IGZO depositions.

**Figure 5:**
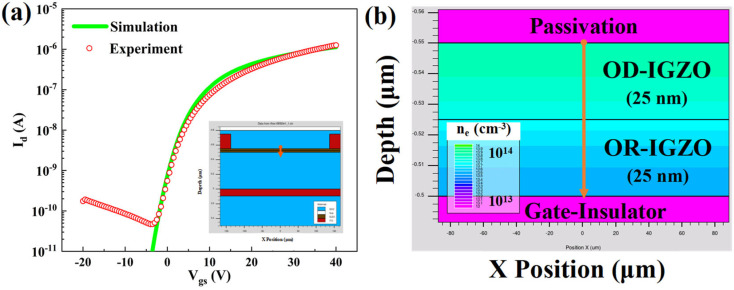
(**a**) The typical transfer curves of the DSCL-TFTs from the simulation results and the experimental data, with the inset being the simulated device structure; (**b**) the simulated electron concentration distribution in the reference device.

Figure 5a shows the fitting results between the simulation data and the experimental transfer curve. With the typical parameters listed in Table 3 and the device built in the inset of Figure 5a, the simulated transfer curve of the DSCL-TFTs agreed well with the experimental data, suggesting that our TCAD model could be used to investigate the physical essence relating the experiments employed in this study. The distribution of the electron concentration (n_e_) in DSCL is exhibited in Figure 5b when V_gs_ = 0 V. Figure 5b shows the detailed simulation results about the area highlighted by a red arrow in the inset of Figure 5a. One may observe that the electron concentration gradually decreased from the back channel to the front channel, where the n_e_ values at the back channel and the front channel were 1 × 10^14^ cm^−3^ and 1 × 10^13^ cm^−3^, respectively. This was consistent with the XPS characterization results shown in Figure 4.

**Figure 6:**
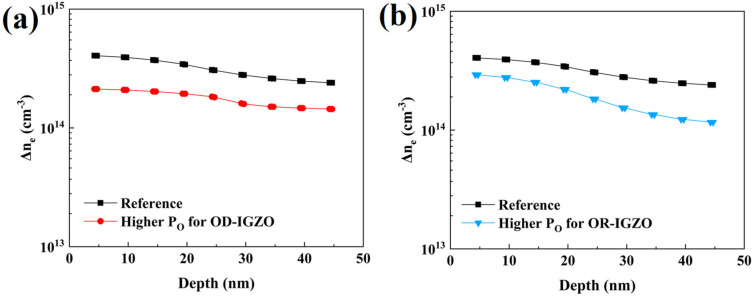
The simulated Δn_e_ distributions in DSCL with varying depth for the cases of (**a**) increasing P_O_ for OD-IGZO and (**b**) increasing P_O_ for OR-IGZO.

After the DSCL-TFTs were illuminated by the 380 nm UV light for 600 s, the electron concentration n_e_ increased to n_e_′. The variation of the electron concentration (Δn_e_ = n_e_′ − n_e_) could microscopically denote the UV light sensing abilities of the DSCL-TFTs. Apparently, the larger the Δn_e_ value, the more sensitive the sample. As shown in Figure 6, Δn_e_ of the reference sample gradually decreased from the back channel to the front channel. The simulation studies exhibited that Δn_e_ apparently decreased by increasing P_O_ either for OD-IGZO (as shown in Figure 6a) or for OR-IGZO (as shown in Figure 6b). This result explained the experimental results shown in Figure 2, i.e., the UV light sensing abilities of the DSCL-TFTs became worse with P_O_ increasing. On the other side, the P_O_ modulation effects from the OD-IGZO and OR-IGZO were different. From the aforementioned experiments, the P_O_ variation for OD-IGZO deposition showed stronger influence than that for OR-IGZO. To more deeply explore this difference, we calculated the total Δn_e_ by summing all the data points shown in Figure 6a,b. As shown in Table 4, the increase in P_O_ for OD-IGZO showed stronger influences on the total Δn_e_ than that for OR-IGZO, which is also consistent with the measurement results shown in Figure 2. It is well known that the electron concentration closely relates oxygen vacancies and the other defects in a-IGZO [27,28], so the simulation results shown in Figure 6 also reflected how the defects (especially V_O_) changed with P_O_ and UV light illuminations. Therefore, for DSCL-TFT UV light detectors, the defects in OD-IGZO (especially the V_O_ density) were more easily modulated by P_O_ than those in OR-IGZO. 

According to all the experimental data and simulation results, we made a deeper discussion on the P_O_ effect for DSCL-TFT UV light response. From the UVS characterization results shown in Figure 3, we could assume that band-to-band electron excitation would be nearly the same for all the devices in this study. On the one hand, the net photogenerated electron concentration (Δn_e_) decreased with P_O_ for DSCL increasing and the photogenerated V_O_^2+^ were believed to play an important role in UV light sensing [22,28]. Therefore, we could suppose that the P_O_ increase during the DSCL depositions might reduce the concentration of photogenerated V_O_^2+^ and result in smaller Δn_e_ in the channel layers. There are two main photogenerated V_O_ creation mechanisms under the UV light illuminations, which could be formed by using the energy released from electron-hole recombination or photogenerated owing to the high energy photons [22]. Therefore, the concentration of photogenerated V_O_^2+^ might decrease when P_O_ for a-IGZO depositions increased; this might be induced by lower photoionization of V_O_ to V_O_^2+^ rather than lower V_O_ creation under the illumination, because the photogenerated V_O_ might hardly be affected by the P_O_ value for a-IGZO. In other words, when the P_O_ value for a-IGZO deposition increased, V_O_^2+^ from the photoionization of V_O_ decreased, resulting in a decrease in Δn_e_ and thus less UV light sensing for the DSCL-TFTs.

Finally, we may reach some design guidelines for DSCL-TFT UV detectors: (1) The DSCL consisting of an OD-IGZO (top side) and an OR-IGZO (bottom side) is the best choice for UV light detection; (2) For the deposition of DSCL, lower oxygen partial pressures are preferred; (3) To modulate the UV light sensing abilities of DSCL-TFTs, the OD-IGZO, rather than OR-IGZO, is more sensitive to variations in P_O_ values.

## 4. Conclusions

The influences of oxygen partial pressures for DSCL-TFTs on their UV light sensing abilities were investigated by both experimental studies and TCAD simulations. With the increase in P_O_ values for the DSCL depositions, the sensing parameters, including I_photo_, S, R, and D* of the corresponding TFTs, apparently became worse. Compared with the P_O_ variations for the OR-IGZO films, those for the OD-IGZO depositions showed stronger influences on the sensing performances of the DSCL-TFT UV light detectors. The TCAD simulations indicated that the variations of the electron concentrations (or the V_O_ density) with P_O_ values under UV light illuminations should be responsible for the aforementioned experimental results. Some design guidelines for DSCL-TFT UV light detectors were finally proposed for the potential applications of these novel semiconductor devices.

## Figures and Tables

**Table 1:** Oxygen partial pressure for the channel-layer depositions of the DSCL-TFTs (Unit: mPa).

	Device 1	Device 2	Device 3	Device 4	Device 5
OD-IGZO	0	7	14	7	7
OR-IGZO	21	21	21	40	56

**Table 2:** Variations of the UV light detecting performances with ΔP_O_ for OD-IGZO and OR-IGZO.

P_O_ Variation Layer	ΔI_photo_/ΔP_O_ (A·mPa^−1^)	ΔS/ΔP_O_ (dB·mPa^−1^)	ΔR/ΔP_O_ (A·W^−1^·mPa^−1^)	ΔD*/ΔP_O_ (Jones·mPa^−1^)
OD-IGZO	−7.5 × 10^−11^	−1.9	−3.2 × 10^−4^	−2.2 × 10^9^
OR-IGZO	−1.9 × 10^−11^	−0.8	−8.2 × 10^−5^	−5.6 × 10^8^

Here, 1 Jones = 1 cm·Hz^0.5^·W^−1^.

**Table 3:** Typical simulation parameters for a-IGZO films in DSCL-TFTs.

Parameter.	OD-IGZO	OR-IGZO	Description
NC300 (cm^−3^)	5.0×10^19^	5.0×10^19^	The conduction band equivalent density at 300 K.
NV300 (cm^−3^)	1.55×10^20^	1.55×10^20^	The valence band equivalent density at 300 K.
EG300 (eV)	3.65	3.65	The energy gap at 300 K.
Affinity (eV)	4.16	4.16	The electron affinity.
NTD (cm^−3^eV^−1^)	3.0×10^21^	3.0×10^21^	The donor-like trap density per unit energy at the valence band edge.
NTA (cm^−3^eV^−1^)	5.9×10^22^	6.1×10^22^	The acceptor-like trap density per unit energy at the conduction band edge.
NGD (cm^−3^eV^−1^)	3.8×10^17^	1.8×10^17^	The donor-like trap density per unit energy at the peak of the Gaussian distribution.
NGA (cm^−3^eV^−1^)	5.9×10^16^	6.5×10^16^	The acceptor-like trap density per unit energy at the peak of the Gaussian distribution.
EGD (eV)	2.70	2.70	The energy level where the Gaussian distribution peaks of donor-like trap.
EGA (eV)	0.70	0.70	The energy level where the Gaussian distribution peaks of acceptor-like trap.
WTD (eV)	0.22	0.22	The characteristic decay energy of donor-like trap exponential tail distribution.
WTA (eV)	0.02	0.02	The characteristic decay energy of acceptor-like trap exponential tail distribution.
WGD (eV)	0.15	0.15	The characteristic decay energy of donor-like trap Gaussian distribution.
WGA (eV)	0.28	0.28	The characteristic decay energy of acceptor-like trap Gaussian distribution.
n(V_O_) (cm^−3^)	5.2×10^16^	4.8×10^16^	Initialized V_O_ concentration.
n(V_O_^2+^) (cm^−3^)	1.7×10^16^	1.3×10^16^	Initialized V_O_^2+^ concentration.
n(I_O_) (cm^−3^)	3.3×10^16^	3.3×10^16^	Initialized I_O_ concentration.
n(I_O_^2−^) (cm^−3^)	1.4×10^5^	1.4×10^5^	Initialized I_O_^2−^ concentration.

**Table 4:** Total net photogenerated electron concentration of the DSCL-TFTs.

	Reference	Higher P_O_ for OD-IGZO	Higher P_O_ for OR-IGZO
Total Δn_e_ (cm^−3^)	5.7 × 10^15^	3.2 × 10^15^	3.5 × 10^15^

## Data Availability

The data presented in this study are available on request from the corresponding author.
